# Mobile Apps to Improve Medication Adherence in Cardiovascular Disease: Systematic Review and Meta-analysis

**DOI:** 10.2196/24190

**Published:** 2021-05-25

**Authors:** Shahd Al-Arkee, Julie Mason, Deirdre A Lane, Larissa Fabritz, Winnie Chua, M Sayeed Haque, Zahraa Jalal

**Affiliations:** 1 Institute of Clinical Sciences University of Birmingham Birmingham United Kingdom; 2 Liverpool Centre for Cardiovascular Science University of Liverpool Liverpool United Kingdom; 3 Institute of Cardiovascular Sciences University of Birmingham Birmingham United Kingdom; 4 Institute of Applied Health Research University of Birmingham Birmingham United Kingdom

**Keywords:** mobile health care applications, medication adherence, cardiovascular disease, systematic review, mobile phone

## Abstract

**Background:**

Adherence rates of preventative medication for cardiovascular disease (CVD) have been reported as 57%, and approximately 9% of all CVD events in Europe are attributable to poor medication adherence. Mobile health technologies, particularly mobile apps, have the potential to improve medication adherence and clinical outcomes.

**Objective:**

The objective of this study is to assess the effects of mobile health care apps on medication adherence and health-related outcomes in patients with CVD. This study also evaluates apps’ functionality and usability and the involvement of health care professionals in their use.

**Methods:**

Electronic databases (MEDLINE [Ovid], PubMed Central, Cochrane Library, CINAHL Plus, PsycINFO [Ovid], Embase [Ovid], and Google Scholar) were searched for randomized controlled trials (RCTs) to investigate app-based interventions aimed at improving medication adherence in patients with CVD. RCTs published in English from inception to January 2020 were reviewed. The Cochrane risk of bias tool was used to assess the included studies. Meta-analysis was performed for clinical outcomes and medication adherence, with meta-regression analysis used to evaluate the impact of app intervention duration on medication adherence.

**Results:**

This study included 16 RCTs published within the last 6 years. In total, 12 RCTs reported medication adherence as the primary outcome, which is the most commonly self-reported adherence. The duration of the interventions ranged from 1 to 12 months, and sample sizes ranged from 24 to 412. Medication adherence rates showed statistically significant improvements in 9 RCTs when compared with the control, and meta-analysis of the 6 RCTs reporting continuous data showed a significant overall effect in favor of the app intervention (mean difference 0.90, 95% CI 0.03-1.78) with a high statistical heterogeneity (I^2^=93.32%). Moreover, 9 RCTs assessed clinical outcomes and reported an improvement in systolic blood pressure, diastolic blood pressure, total cholesterol, and low-density lipoprotein cholesterol levels in the intervention arm. Meta-analysis of these clinical outcomes from 6 RCTs favored app interventions, but none were significant. In the 7 trials evaluating app usability, all were found to be acceptable. There was a great variation in the app characteristics. A total of 10 RCTs involved health care professionals, mainly physicians and nurses, in the app-based interventions. The apps had mixed functionality: 2 used education, 7 delivered reminders, and 7 provided reminders in combination with educational support.

**Conclusions:**

Apps tended to increase medication adherence, but interventions varied widely in design, content, and delivery. Apps have an acceptable degree of usability; yet the app characteristics conferring usability and effectiveness are ill-defined. Future large-scale studies should focus on identifying the essential active components of successful apps.

**Trial Registration:**

PROSPERO International Prospective Register of Systematic Reviews CRD42019121385; https://www.crd.york.ac.uk/prospero/display_record.php?RecordID=121385

## Introduction

Cardiovascular diseases (CVDs) are responsible for almost one-third of all deaths worldwide, leading to an estimated 17.9 million deaths each year [1]. A long-term use of cardiovascular medications significantly reduces the risk of morbidity and mortality [2,3], but their full therapeutic potential cannot be achieved if patients are nonadherent [4]. Approximately 9% of all CVD events in Europe are attributed to poor medication adherence [5], with adherence rates of only 57% [6].

Developing interventions to tackle medication nonadherence is important for improving health outcomes. A recent network meta-analysis of many different interventions showed that those with a technology-based approach had a positive, but short-lived, effect on medication adherence [7]. The escalating inclusion of technology into everyday life has witnessed the introduction of mobile health (mHealth) interventions, such as mobile apps, to support patients and health care professionals (HCPs) in disease management [8,9]. These reviews were not confined to app-based interventions. Some of the wide-ranging interventions included apps, whereas other mHealth interventions, such as text messaging and emails, were common. Several systematic reviews have indicated that apps may play a role in improving medication adherence in patients with CVD. For example, one systematic review included smartphone app-based interventions to promote lifestyle and behavior changes, reporting them as effective at improving medication adherence and increasing physical activity behavior [10]. For secondary prevention in patients with cerebrovascular disease, another systematic review showed improved medication adherence, a better maintenance of blood pressure (BP) and lipids within target ranges, and decreased episodes of angina, transient ischemic attack, and stroke with mHealth interventions, several of which included apps [11]. In contrast, a systematic review of internet-based interventions, which included apps, improved dietary outcomes, quality of life (QoL), and physical activity but reported a lack of evidence for their effect on medication adherence [12].

Published evidence for the beneficial effects of apps on medication adherence is often lacking or inconclusive. This study evaluates the effectiveness of app-based interventions on medication adherence in patients with CVD. Furthermore, this study explores the effects of app-based interventions on health-related outcomes, the functionality and usability of apps for patients, and the involvement of HCPs in the delivery of the intervention.

## Methods

### Search Strategy and Study Selection

This review followed the PRISMA (Preferred Reporting Items for Systematic Reviews and Meta-Analyses) guidelines [13]. The protocol was registered in the PROSPERO database (CRD42019121385) [14].

MEDLINE (Ovid), PubMed Central, Cochrane Library, CINAHL Plus, PsycINFO (Ovid), Embase (Ovid), and Google Scholar databases were searched from inception to January 2020 using a 3-domain search strategy to include terms related to CVD, apps, and medication adherence (the search strategy is presented in Multimedia Appendix 1). Studies were selected if they were randomized controlled trials (RCTs), if they were published in English, if they were for patients with CVD (eg, atrial fibrillation, coronary heart disease, heart failure, hypercholesterolemia, hypertension, myocardial infarction, and stroke), and if the intervention included an app to improve medication adherence. A 2-stage process was used to select studies for inclusion in this review. First, 1 author (SA) screened titles and abstracts for relevance and removed duplicate records. Where ambiguities arose from the screening process, 1 of 2 different authors (JM and ZJ) independently assessed the title and abstract for relevance. For the second stage of screening, 2 authors (SA and ZJ) independently reassessed the full-text studies matching the prespecified criteria for eligibility. Bibliographies of selected studies were hand searched for additional references.

### Data Extraction and Quality Assessment

Data extraction was conducted using a standardized form developed specifically for this review. Extracted data included the characteristics of the study and details of the intervention strategy. Quality assessment was conducted independently by 2 authors (SA and ZJ), and consensus was reached through discussion. The risk of bias was guided by the Cochrane Collaboration tool for RCTs [15], with the bias broadly categorized as selection, performance, attrition, or other bias. Once categorized, each bias domain was further categorized as low, high, or unclear risk of bias. Agency for Healthcare Research and Quality standards [15] were then applied, and an overall summary was generated using Review Manager (RevMan. version 5.4, The Cochrane Collaboration) [16].

### Data Synthesis and Statistical Analysis

The outcome data were extracted from each trial. The authors were contacted for raw data where follow-up points for individual trials were identified but outcomes not reported in the published manuscripts. Four main analyses were conducted: (1) a series of meta-analyses of intervention effects on medication adherence at different time points of intervention duration; (2) a univariable meta-regression analysis, regressing the app intervention across trials on intervention duration; (3) a meta-analysis of intervention effects on medication adherence across all included trials at the final time point of intervention duration; and (4) a series of meta-analyses of intervention effects on systolic blood pressure (SBP), diastolic blood pressure (DBP), total cholesterol (TC), and low-density lipoprotein cholesterol (LDL-C) levels at the third month of the intervention. For the meta-analyses, trials reporting continuous data, means, SD, and sample sizes were included. Where SE or CI were reported, the SD was manually calculated. A random-effects model was used to allow for differences in the true intervention effect across trials. The Q test was used to assess heterogeneity, with a significant result (*P*<.05) indicating heterogeneity across trials. The I^2^ statistic was computed to describe the percentage of variability effect estimates due to heterogeneity. I^2^ values of 25%, 50%, and 75% were assigned as low, moderate, and high heterogeneities, respectively [17]. The statistical package STATA (StataCorp, Stata Statistical Software: Release 16) was used for the meta-analysis [18].

## Results

### Search Results

**Figure 1:**
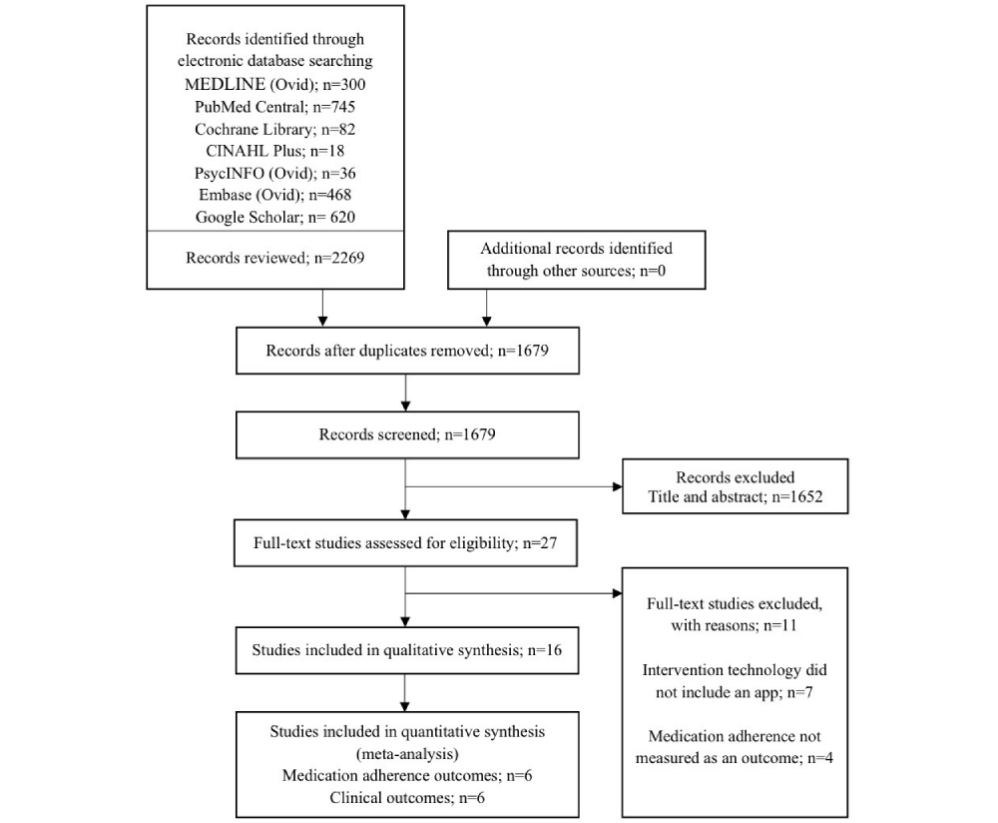
PRISMA (Preferred Reporting Items for Systematic Reviews and Meta-Analyses) flow diagram depicting study selection.

Searches yielded 2269 citations, of which 590 duplicates were removed. The title and abstract screening resulted in 27 full-text review studies. Of these, 11 studies were excluded. No additional citations were identified by hand searching. Therefore, 16 RCTs were included in this review [19-34]. A PRISMA ﬂowchart summarizing the study selection is shown in Figure 1.

### Study Characteristics and Design

All the included studies were published between 2013 and 2019. They all compared 1 or more interventions (app alone or app in conjunction with a package of participant support) with a control arm described as usual care. A total of 10 studies randomized patients to parallel intervention or control groups [20,21,23,25-28,31-33]; 2 had a crossover design [19,30]; and 4 were cluster randomized by the trial site [22,24], researcher [29], or physician [34]. Study sample sizes ranged from 24 [30] to 412 [31], and interventions ranged in duration from 1 [23,30,32] to 12 [29] months. The definition of usual care in the control groups differed among the studies. It was defined as follow-up without the use of the app in 7 trials [22,24,26,27,29,31,33]; the use of the app with limited functionality in 1 trial [32]; and an alternative intervention not including apps, for example, the use of a SMS text message [20,28,34], follow-up phone calls [21], use of a pillbox [23], and use of an e-diary [25] in 6 trials. For the 2 crossover trials, nondigital technology methods were used [19,30] (Table 1).

**Table 1:** Characteristics of the included randomized controlled trials.

Source; country	RCT^a^ design	Number of randomized participants	Intervention and control arm	Length of intervention	Primary and secondary outcomes measures
Brath et al [19]; Austria	Crossover 2-arm	77	Intervention: monitoring phase using app Control: control phase using paper diary	20 weeks	Primary: medication adherence Secondary: changes in SBP^b^, DBP^c^, HbA1c^d^, LDL-C^e^, and usability of the app
Chandler et al [20]; United States	Parallel 2-arm	54	Intervention: SMASH^f^ app Control: SMS text messages on lifestyle tips unrelated to medication adherence	9 months	Primary: change in SBP Secondary: change in DBP, medication adherence, and patients’ satisfaction with using the app
Fang and Li [21]; China	Parallel 3-arm	280	Intervention: 2 arms: (1) SMS text messages using an app and (2) SMS text messages using an app plus micro letter Control: phone	6 months	Primary: medication adherence Secondary: none
Frias et al [22]; United States	Parallel 3-arm, clustered by study site	118	Intervention: a DMO^g^ system designed to provide feedback about taking medication to both patients and providers consisted of an ingestible sensor, sensor patch, and app, 2 arms: (1) 4-week DMO and (2) 2.12-week DMO Control: no system use	12 weeks	Primary: change in SBP Secondary: changes in SBP, DBP, HbA1c, and LDL-C; medication adherence; and satisfaction with using the app
Goldstein et al [23]; United States	Parallel 2×2 arm	60	Intervention: 2 arms: (1) mHealth^h^ app reminder and (2) mHealth app silent Control: 2 arms: (1) telehealth (pillbox reminder) and (2) telehealth (pillbox silent)	28 days	Primary: medication adherence Secondary: acceptance of the app
Guo et al [24]; China	Parallel 2-arm, clustered by study site	209	Intervention: mAF app Control: no app use	3 months	Primary: medication adherence, usability of the app, PAM^i^, patients’ knowledge, anticoagulation satisfaction, and QoL^j^ Secondary: none
Johnston et al [25]; Sweden	Parallel 2-arm	174	Intervention: interactive patient support tool (web-based app) Control: no app use, only simplified tool	6 months	Primary: medication adherence Secondary: change in SBP and LDL-C; QoL; and usability of the app
Kim et al [26]; Republic of Korea	Parallel 2-arm	95	Intervention: Wireless Self-Monitoring, an app with enrolled in the HealthyCircles Platform Control: no app use	6 months	Primary: medication adherence, PAM, SBP, and DBP Secondary: none
Labovitz et al [27]; United States	Parallel 2-arm	28	Intervention: artificial intelligence app Control: no daily monitoring	12 weeks	Primary: medication adherence Secondary: medication adherence for patients receiving DOACs^k^ and usability of the app
Liu et al [28]; China	Parallel 2-arm	57	Intervention: HeartGuardian app and weekly text messages on health education Control: weekly SMS text messages on health education	12 weeks	Primary: HDL-C^l^, LDL-C, TC^m^, and triglyceride Secondary: medication adherence
Márquez Contreras et al [29]; Spain	Parallel 2-arm, clustered by researchers	154	Intervention: AlerHTA app Control: usual care in arterial hypertension	12 months	Primary: medication adherence Secondary: SBP and DBP
Mertens et al [30]; German	Crossover 2-arm	24	Intervention: medication app Control: a paper diary	28 days	Primary: medication adherence Secondary: user experience of the app
Morawski et al [31]; United States	Parallel 2-arm	412	Intervention: Medisafe app Control: no intervention	12 weeks	Primary: medication adherence and change in SBP Secondary: SBP and DBP
Ni et al [32]; China	Parallel 2-arm	50	Intervention: BB app and WeChat app Control: WeChat app	30 days	Primary: medication adherence and heart rate, SBP, and DBP Secondary: acceptability of the app
Santo et al [33]; Australia	Parallel 3-arm	166	Intervention: 2 arms: (1) basic medication reminder app and (2) advanced medication reminder app Control: standard care as determined by patients’ physicians	3 months	Primary: medication adherence Secondary: BP^n^, TC, LDL-C, and acceptability of the app
Sarfo et al [34]; Ghana	Parallel 2-arm, clustered by physician	60	Intervention: Blue-toothed UA-767Plus BT BP device and a smartphone with an embedded app Control: SMS text messages on healthy lifestyle behaviors	3 months	Primary: BP Secondary: medication adherence, hypertension management competence, autonomous self-regulation score for glucose control, patients’ satisfaction with using the app, side effects of antihypertensive medications, hypertension, and stroke knowledge

^a^RCT: randomized controlled trial.

^b^SBP: systolic blood pressure.

^c^DBP: diastolic blood pressure.

^d^HbA_1c_: glycated hemoglobin.

^e^LDL-C: low-density lipoprotein cholesterol.

^f^SMASH: Smartphone Med Adherence Stops Hypertension.

^g^DMO: digital medicine offering system.

^h^mHealth: mobile health.

^i^PAM: patient activation measure.

^j^QoL: quality of life.

^k^DOAC: direct oral anticoagulant.

^l^HDL-C: high-density lipoprotein cholesterol.

^m^TC: total cholesterol.

^n^BP: blood pressure.

### Participant Characteristics

The included trials covered a range of different CVDs and risk factors: atrial fibrillation [24], coronary heart disease [21,30,32,33], diabetes [19,22], heart failure [23], hypercholesterolemia [19], hypertension [19,20,22,26,29,31], myocardial infarction [25,28,30], and stroke [27,34]. The mean age of participants varied depending on the disease and ranged from 46.5 (SD 9.9) [20] to 73.8 (SD 7.5) years [30]. All studies recruited outpatients from secondary care [19,21,23-28,30,32,34], primary care [20,22,29], tertiary care [33], or web-based patient communities [31].

### App Characteristics

The characteristics of the trialed apps are shown below. Each study used a different app developed by different organizations: 8 were academic or government institutions [20,21,23,24,26,28-30], whereas others were commercial organizations. A total of 7 apps were supported by platforms [19-22,24,26,27]. The functionality of the apps and platforms varied across the different trials and the interactions needed by patients. All but 2 apps [24,26] delivered medication reminders to promote medication adherence. For the majority, this was their primary function, with 4 apps using one-way SMS text message reminders [21,27,28,32] and 5 delivering a mobile device alert [23,29-31,33]. Others had a primary focus on self-monitoring alone [26] or with a medication reminder [25], patient education [24], or delivery of a tailored motivational SMS text message based on medication adherence levels [20,34]. Two trials used the app to transmit patients’ adherence data to the associated platform to be monitored by HCPs [19,22].

### Involvement of HCPs

Half of the trials involved physicians and/or nurses in app use [19,21,24,25,29,30,32,34] (Table 2). A trial involved pharmacists in blinding study medication, whereas the health care team, whose professions remained unspecified, used the app and monitored the data transmitted to the associated platform [22]. One trial permitted the sharing of patients’ data with families and caregivers as well as with HCPs [26]. A total of 6 trials did not specify the type of HCPs involved in app use [20,23,27,28,31,33]. The involvement of HCPs varied; most of the trials involved HCPs to monitor patients’ data [19,22,24-26,30,34], instruct patients on how to use the app [29], and send educational materials to patients via the app [21,32].

**Table 2:** Mobile app characteristics in the included randomized controlled trials.

Source	App name and functionality	Platform used with the app and functionality	Overall system functionality	Involvement of HCP^a^
Brath et al [19]	Name not specified, referred to as a mobile phone–based data gateway. Reader and transmitter of data from electronic medication blister to a remote database	Remote telemonitoring service: data sent from the app to platform and then analyzed for timing and number of pills taken, and an automatic reminder is sent to patients via SMS text messages	Reminder	Physician
Chandler et al [20]	SMASH^b^ app: medication reminders via signals (blinking light, intermittent chime, automated SMS text messages, or phone call) and BP^c^ monitor reminders via SMS text messages. The app provided timely tailored motivational and reinforcement SMS text messages based on the levels of medication adherence and SMS text message reminders to monitor BP with a Bluetooth-enabled BP device. The app also provided a cumulative table of average BP displayed in categories of daily, weekly, and/or monthly progress reports	HIPAA^d^-compliant servers: BP data sent from the app to platform, then analyzed for processing with timestamps, providing information for the calculation of adherence levels to the BP protocol	Reminder	Not stated
Fang and Li [21]	Name not specified, referred to as a messaging app: medication reminders via an SMS text messaging app, educational materials via micro letter	Huaxi-gold card: the platform sent SMS text messages, images, media content related to disease and other information at regular intervals	Reminder and education	Physician and nurse
Frias et al [22]	Proteus Discover app: reader and transmitter of the patient’s adherence data from patch to the cloud and prompted the patient to take their medication doses as scheduled. Patients could visualize their data on their mobile devices via the app	Provider web portal: provider views summaries of the DMO^e^ data for the patients on the web portal	Reminder and education	Clinic staff, pharmacist had a role in set up (coencapsulation of ingestible sensor and medication)
Goldstein et al [23]	Name not specified, referred to as a medication adherence app. Medication reminders provided via alert, patients could view list of medications with instructions, and they were able to record taking their medication	No platform	Reminder and education	Not stated
Guo et al [24]	mAF app: educational app used by both patients and physicians: For patients, personal health record (CHA_2_DS_2_-VASc^f^, HAS-BLED^g^, and SAMe-TT_2_R_2_^h^ scores), patient educational programs (knowledge of atrial fibrillation and learn how to manage themselves at home), patient involvement in self-care items (monitor their heart rate, BP, and their quality of life), and structured follow-up consultation via a sent alert reminder. For physicians, clinical decision support	Cloud platform: data management	Education	Physician
Johnston et al [25]	Name not specified, referred to as an interactive patient support tool app: medication reminders via SMS text messages (e-diary) to register daily ticagrelor intake. Secondary prevention educational modules (exercise module, BMI module, and BP module)	No platform	Reminder and education	Physician and nurse
Kim et al [26]	HealthyCircles: an educational app that allowed patients and nurses to access the patient’s reading recorded on the BP monitor devices. The BP measurements are wirelessly uploaded from BP devices to the HealthyCircles account	HealthyCircles platform: the platform sent reminders for self-monitoring BP, education information about the disease condition, and general health behavior recommendations	Education	Families, caregivers, and HCPs (profession not specified)
Labovitz et al [27]	Artificial intelligence app: medication reminders and dosing instructions via SMS text messages. Late doses generated notifications within the hour and before the end of the dosing window	Artificial intelligence platform: the platform sent an automatic SMS text message or emails to clinical staff if doses were missed, late, or based on incorrect use	Reminder	Clinic staff (profession not specified)
Liu et al [28]	HeartGuardian app: medication reminders via SMS text messages. The app provided educational materials; medication recording and daily feedback; and self-empowerment via automatic intelligent, real-time video feedback based on the subjects’ medication adherence	No platform	Reminder and education	Not stated
Márquez Contreras et al [29]	AlerHTA app: medication and appointments reminders via alerts. The app recorded patients’ personal data, the physician’s advice about the prescribed treatment, and the results of the BP measurement. The app recommended BP levels as objectives	No platform	Reminder and education	Physician
Mertens et al [30]	iNephro medication plan app: medication reminders via alert, to support the drug intake needs of patients with chronic conditions on polypharmacy	No platform	Reminder	Physician
Morawski et al [31]	Medisafe app: medication reminders via alert. The app provided alerts to remind patients when it is time to take medications and generate weekly adherence reports, the app also allowed for tracking of BP and other biometric measurements	No platform	Reminder	Not stated
Ni et al [32]	BB reminder app and WeChat app: medication reminders via SMS text messages through the BB reminder app. Educational materials through the WeChat app	No platform	Reminder and education	Physician and nurse
Santo et al [33]	No specified name. Referred to as a medication reminder app. Medication reminders provided via alert. In the basic app, the reminders were noninteractive and occurred 1 time only, whereas the advanced app provided interactive and customizable features including daily reminders, which could be snoozed, rescheduled, and/or marked as a taken or missed dose; medication refill reminders; adherence statistics; and ability to share information with others such as family members, if the patient missed a medication dose	No platform	Reminder	Not stated
Sarfo et al [34]	No specified name. Referred to as medical regimen assistance app. Medication reminders provided via SMS text messages. The app reported BP measurements and medication intake and sent written and oral information on adherence criteria to take the medications within 2 hours of designated times and to measure BP every 3 days in the morning and evening	No platform	Reminder	Nurse

^a^HCP: health care professional.

^b^SMASH: Smartphone Medication Adherence Stops Hypertension.

^c^BP: blood pressure.

^d^HIPAA: Health Insurance Portability and Accountability.

^e^DMO: digital medicine offering system.

^f^CHA_2_DS_2_-VASc: congestive heart failure, hypertension, age>75 years (doubled), type 2 diabetes mellitus, previous stroke, transient ischemic attack or thromboembolism (doubled), vascular disease, age of 65-75 years, and sex.

^g^HAS-BLED: Hypertension, abnormal renal or liver function, stroke, bleeding history or predisposition, labile international normalized ratio, age>65 years, drugs or alcohol concomitantly.

^h^SAMe-TT_2_R_2_: sex, age, medical history, treatment, tobacco use, and race.

### Assessment of Medication Adherence

Adherence measures varied among studies (Multimedia Appendix 2 [19-34]). Most used questionnaires to include the validated 8-item Morisky Medication Adherence Scale [20,26,31,33] and the 4-item Morisky Medication Adherence Scale [21,28] and nonvalidated self-report questionnaires [24,25,30,32]. Other adherence measures included medication event monitoring systems (MEMSs) [29] and a digital medicine offering with an ingestible sensor taken alongside medication [22]. Other trials used a combination of measures; 2 trials combined 2 different measures, one for each arm. One trial used the remote medication adherence measurement system for the intervention and pill count for the control [19]. Another trial used an electronic self-report for the intervention and pillbox openings for the control [23]. Only 2 trials combined 2 different measures for both arms (pill counts and plasma samples [27] and pill counts and 8-item Morisky Medication Adherence Scale [34]).

### Effect on Medication Adherence

**Figure 2:**
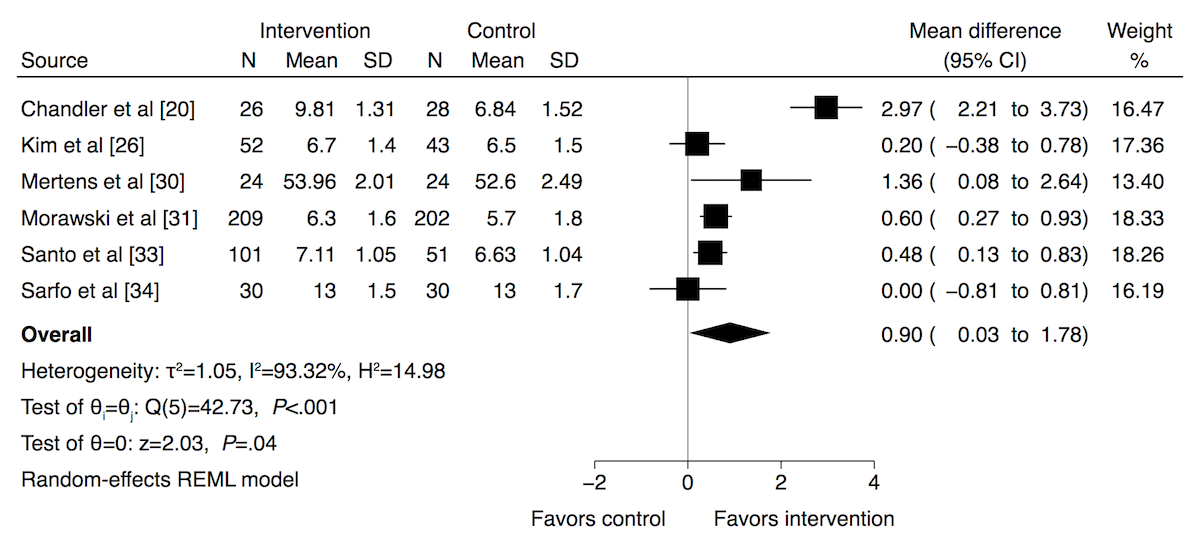
Meta-analysis results and forest plot of the effect of app-based interventions on medication adherence. Mean difference (95% CIs) are denoted by black boxes (black lines). The combined mean difference estimate for all studies is represented by a black diamond, where diamond width corresponds to 95% CI bounds. REML: restricted maximum likelihood.

Overall, 12 trials reported apps to enhance medication adherence rates [20,21,24,25,27-34], with 9 demonstrating significant improvement [20,24,25,28-31,33,34]. In the remaining 4 trials, 3 did not find a significant difference [22,23,26] and 1 reported a significant difference, with only 1 of the 4 medicines being monitored [19] (Multimedia Appendix 2 [19-34]). Six trials reporting continuous data were included in the meta-analysis of medication adherence [20,26,30,31,33,34]. Trials with the same duration of follow-up for the intervention were subjected to a separate meta-analysis and all favored the intervention, mean difference for month 1, 1.52 (95% CI 0.89 to 2.15); 2 trials [20,30] for month 3, 0.46 (95% CI 0.21 to 0.71); 4 trials [20,31,33,34], for month 6, 1.46 (95% CI −1.02 to 3.95); 2 trials [20,26], and for month 9, 1.49 (95% CI −1.42 to 4.40); 2 trials [20,34]. Meta-regression analysis for these 6 studies showed that the duration of intervention (ie, the follow-up month) did not exert a statistically significant impact on the effect of the app on medication adherence (*P*=.65). Thus, a combined meta-analysis (Figure 2) over the different trial durations was performed, thereby demonstrating a significant effect in favor of the app intervention (mean difference 0.90, 95% CI 0.03 to 1.78) with a high statistical heterogeneity (I^2^=93.32%).

### Effect on Other Nonclinical Outcomes

An array of nonclinical outcomes was measured across the trials. Two trials have reported patient activation measures (PAMs) [22,26]. One trial reported a higher increase in PAM scores mean change for the intervention arm 7.9 (SE 2.4) when compared with control 1.7 (SE 3.3); mean difference 6.2 (SE 4.6), (95% CI −2.8 to 15.2) [22]. However, for the other trial, there was no significant difference in the average PAM score over the trial period (baseline: 78.0; end of trial: 76.0; *P*=.34) [26]. Patients’ knowledge of their CVD was only reported in 2 trials [24,34], despite 9 of the 16 trials involving apps with an educational function [21-26,28,29,32]. Interestingly, one trial showed not only a significant improvement in knowledge with app use but also in medication adherence [24]. In the same trial, the benefits and burden of anticoagulation therapy were explored using a patient satisfaction questionnaire. Patients using the app expressed more anticoagulant *benefits,* whereas the control declared more *burden*: benefit (intervention: mean 15.6, SD 2.73 vs control: mean 14.21, SD 3.37; *P*=.05) and burden (intervention: mean 15.57, SD 6.57 vs control: mean 19.30, SD 6.39; *P*=.008) [24]. In the other trial, the knowledge questionnaire scores increased at the end of the trial but not significantly (intervention: mean 10.8, SD 0.8 vs control: mean 11.1, SD 1.1; *P*=.23) [34]. The QoL was assessed in 2 trials using the European Quality of Life–5 Dimensions measure [24,25]. One trial reported signiﬁcantly higher QoL in the intervention arm compared with the control (*P*<.05; exact *P* value not quoted in original paper) [24], whereas in the other trial, QoL scores increased with app use over the duration of the trial but not significantly (*P*=.06) [25].

### Effect on Clinical Outcomes

Clinical outcomes measured included BP, blood cholesterol, and blood glucose (Multimedia Appendix 3 [19,20,22,25,26,28,29,31-34]). Eight trials reported positive effects of apps on both SBP and DBP [19,20,22,25,26,29,33,34], and 4 reported significant results [19,20,22,29]. In total, 4 trials reported improvements in TC [19,22,28,33] and 3 were significant [19,22,28]. A reduction in LDL-C was observed with app-based interventions [19,22,25,28,33], but it was only significant in 2 trials [22,25]. Only 2 trials reported glycated hemoglobin (HbA_1c_) as an outcome, with no significant change [19,22]. Meta-analysis for clinical outcomes was only possible at 3 months duration of intervention for SBP, DBP, TC, and LDL-C; all favored the use of an app in disease management, but not all were significant (Multimedia Appendix 4 [22,25,28,31,33,34]). Meta-analysis for HbA_1c_ was not possible because of the lack of reported outcomes.

### App Usability, Acceptability, and Patient Satisfaction

Various questionnaires were used to evaluate the app usability for patients, but this was only done in 4 trials [19,24,25,27]. One study used a validated System Usability Scale to demonstrate greater usability in the app intervention arm than in the control arm (intervention: mean 87.3, SD 13.9 vs control: mean 78.1, SD 18.9; *P*=.001) [25]. Three trials evaluated app usability with nonvalidated questionnaires and obtained positive feedback from 80% or more of the participants [19,24,27]. Patients with stroke rated the app *extremely good* as a medication management tool and as means to improve physician-patient rapport [27]. Patients with atrial fibrillation agreed that the study app was user-friendly and helpful with additional positive feedback from physicians [24].

Four different trials explored app acceptability in patients [23,30,32,33]. Acceptance rates measured by nonvalidated questionnaires found the app to be more acceptable than the control [23], and most patients reported that the app was useful and helpful [33]. Interviews conducted within 2 studies revealed that patients accepted and appreciated receiving reminders and educational materials via the app [32] and that most patients (22/24) reported wanting to use the app in everyday life [30]. Three trials evaluated patient satisfaction with the apps being trialed by nonvalidated questionnaires, with more than 90% reporting the app as easy to use [20,22,34].

### Risk of Bias of Included Trials

**Figure 3:**
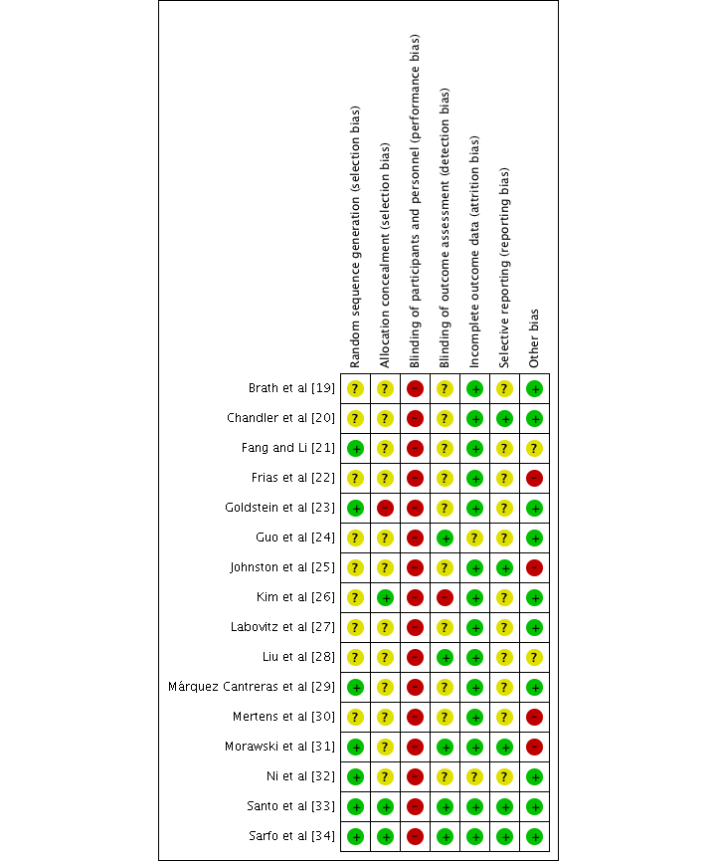
Authors’ judgments about each risk of bias item for each included trial. Green: low risk of bias; yellow: unclear risk of bias; red: high risk of bias.

**Figure 4:**
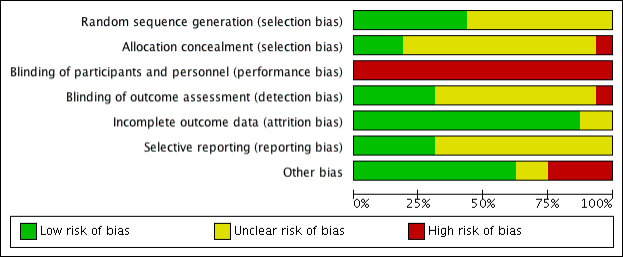
Authors’ judgments about each risk of bias item presented as percentages across all included trials.

Only 7 trials reported sufficient random sequence generation [21,23,29,31-34], and only 3 trials reported allocation concealment [26,33,34]. Although these types of interventions are problematic to blind, outcome assessors could have been blinded, but only 5 trials clearly stated that this was done [24,28,31,33,34]. In total, 14 trials had a low risk of incomplete outcome data [19-23,25-31,33,34], whereas only 5 had a low risk of selective outcome reporting [20,25,31,33,34]. Ten trials had no other sources of risk of bias [19,20,23,24,26,27,29,32-34]. According to the Agency for Healthcare Research and Quality standards, most trials were considered to be of poor quality [19-32], with only 2 rated as fair [33,34]. Figures 3 and 4 present the risk of bias judgment.

## Discussion

### Principal Findings

This study included 16 RCTs that assessed the effectiveness of mobile app–based interventions on medication adherence [19-34]. A total of 9 trials showed a statistically significant improvement in medication adherence in the intervention arm [20,24,25,28-31,33,34]. The meta-analysis of 6 trials revealed that app interventions exert a significant positive effect on medication adherence with meta-regression, showing no statistically significant impact for the duration of use over a maximum of 9 months. However, the statistical and methodological heterogeneity was high [20, 26, 30, 31, 33, 34]. Ten trials assessed health-related outcomes and generally reported an improvement with intervention over control [19,20,22,24-26,28,29,33,34]. The apps used had mixed functionality, including reminders [19,20,27,30,31,33,34], education [24,26], or both [21-23,25,28,29,32]. Regarding the involvement of HCPs, most involved physicians and/or nurses [19,21,24,25,29,30,32,34]. The usability of apps was mainly assessed with questionnaires, with most participants reporting acceptance and ease of use [19,24,25,27]. The effectiveness of app interventions could not be assigned to particular app components or characteristics. Half of the trials were small-scale studies, that is, pilot studies [19,22,24,34] and feasibility studies [21,23,27,32], and most trials were classified as having poor quality of evidence because of the high risk of bias or insufficient reporting of information [19-32].

### Relationship With Previous Published Literature

Previous systematic reviews have assessed the effectiveness of health care apps in the management of several different long-term conditions, including asthma [35], obesity and diabetes [36], and CVD [37]. Most included small-scale studies, with insufficient or low-quality evidence to support app use. Despite this, many reviews have reported beneficial trends, for example, in the promotion of positive behavior changes such as medication adherence [37].

A network meta-analysis of different interventions showed that technology-based interventions exert a major effect on the long-term management of medication adherence in patients with CVD [38]. The World Health Organization categorizes medication adherence measurements as either subjective or objective [39]. More than half of the trials in this systematic review used subjective self-report questionnaires to measure medication adherence [20,21,24-26,28,30-33], with a potential to overestimate adherence. Although there is no gold standard measure of medication adherence, a multi-measure approach is highly recommended to reduce subjectivity [40]. Therefore, the results of improved adherence from the trials included in this review should be interpreted with caution.

This review shows that objective measures can be improved with expected app use. For example, some of the trials included in this review assessed BP and showed improvements for participants in the intervention arms [19,20,22,25,26,29,33,34], a similar result to a previous systematic review assessing the effects of mobile apps designed for BP management [9]. Another systematic review and meta-analysis of 21 RCTs showed a reduction in HbA_1c_ levels in patients with diabetes [41]. In this review, the effectiveness of apps to support patients with diabetes was inconclusive, as only 2 included trials evaluated HbA_1c_, and both the trials reported no significant difference in the change in HbA_1c_ between the intervention and control arms [19,22].

Although few trials included in this review investigated nonclinical outcomes other than adherence, those that did demonstrated a meaningful, but not always significant, improvement in PAM [22,26], patients’ disease knowledge [24,34], anticoagulation satisfaction [24], and QoL [24,25]. These results align with existing systematic reviews of smartphone-based health care technologies, which demonstrate that apps could play an important role in patient education, self-management, and remote monitoring [42] and improvements in patients’ QoL [37]. Furthermore, 2 pilot studies examining the feasibility of app use to enhance safe anticoagulation therapy and knowledge acquisition by patients showed a significant increase in anticoagulation knowledge after 3 months of app use [43,44]. The beneficial effects of apps on medication adherence will likely depend on the nature of the support needed by different patients. To improve medication adherence, the literature suggests that some patients may need only reminders, whereas others need a greater knowledge and understanding of their disease and the medication prescribed [45,46]. There is a long history of reminders and patient education to improve medication adherence, and the introduction of app technology has seen these strategies incorporated into mHealth interventions. In this review, most of the included trials used apps with mixed functionality, including reminders, education, or both. All but 2 of the apps [24,26] included reminders [19-23,25,27-34]; of these, significant improvements in medication adherence were only reported in about half of the trials [20,24,25,28-31,33,34]. Thus, it remains impossible to assign success to a single component within a multifunctional intervention.

App design, user interface, and evaluation of these factors are often under-reported. In this review, 4 trials that assessed app usability demonstrated that the apps were user-friendly, and users were interested and engaged with the technology [19,24,25,27]. Three of these studies featured commercially developed apps [19,25,27]. The measures of app success for developers of commercial, academic, or government origins may explain why only 1 app developed by an academic institution [24] investigated usability. A systematic review of app usability in patients with diabetes also reported moderate to good usability, but users expressed preference for apps developed for tablet computers rather than smartphones due to their larger display and better illustrations [47]. Usability is a key factor in the uptake of mHealth apps [48,49], and it would make sense to conclude that a more user-friendly app might be more effective. In this review, significant improvements in medication adherence rates were found in only 2 of the 4 trials reporting a good usability [24,25]. This may, in part, be because usability outcomes measure ease of use (ie, user-friendliness) rather than motivation, engagement, and continued use. Motivating components, such as social contracts with family members and gamification, have been incorporated into some apps to improve their effectiveness [43]. Several studies highlight the importance of using theory to develop and design behavioral change interventions [50-52], which should also be considered in mHealth app intervention design. Only 3 of the trials in this review [20,28,34], reported the use of behavioral change theories to inform their app intervention, and it is of note that only one of the app interventions purported to involve social support or interactions outside of HCPs [26]. This review revealed that HCPs’ involvement in app interventions for CVD health care mainly involved physicians and nurses [19,21,24,25,28-30,32,34], with 1 trial reporting pharmacist involvement; however, that did not include the administration of the app intervention [22]. With the widening clinical patient-facing roles of pharmacists within primary care [53,54] and reports of their effectiveness in both CVD management [55-57] and successful efforts to improve CVD medication adherence [58,59], it is potentially surprising that pharmacists were not more involved in any of these studies. The involvement of any HCP in the administration and concomitant use of apps with patients requires careful consideration. Such apps have the potential to increase HCP workload, and it remains unclear whether the cost of that involvement outweighs the benefits observed. Of the RCTs included in this review, 5 of the 9 that included HCPs in the administration of the app reported significant improvements in medication adherence, but no cost-benefit analysis was conducted [24,25,29,30,34]. In the current climate, with a growing choice of apps, a more important role for HCPs may be in the recommendation of safe, user-friendly, and effective mHealth apps for patients depending on their disease and apps chosen specifically to meet their patients’ needs and motivations.

### Strengths and Limitations

This review did not consider the differences in adherence between the medications included in the trials. Some medications might have a higher rate of nonadherence than others because of the adverse effects and taste of the formulation. The heterogeneity of the trials’ methodologies, apps, and outcome measures studied made quantitative comparisons problematic. Different measures of medication adherence were used among the trials, which made it impossible to calculate the exact adherence rates. For several of the included trials, control groups were also subjected to an intervention aimed at improving medication adherence, meaning that the impact of the app intervention was not comparable with standard care. This, coupled with the potential for wide variations in standard care more generally, suggests that the findings of many of the included studies need to be interpreted with caution. Finally, this review included only RCTs; thus, other relevant studies and reports from the gray literature were excluded. However, RCTs are considered the cornerstone of clinical research to determine the efficacy of interventions and the highest level of evidence.

### Implication for Practice and Policy

Health care apps have the potential to enhance medication adherence, leading to improvements in clinical and nonclinical outcomes in patients with CVD. However, the use of this technology to support medication adherence is in its infancy, and apps require robust testing to demonstrate its effectiveness. The trials included in this review provided inconsistent data regarding their effectiveness. Overall, user engagement and usability were rated positively, demonstrating interest in the concept. However, it is difficult to make strong, unrestricted recommendations for practice, especially with the methodological limitations of the included trials.

### Implication for Research

This review indicates the need for further large-scale studies to determine whether mobile apps are effective in improving medication adherence among patients with CVD. There is a paucity of data to differentiate the effects of individual app intervention characteristics on behavioral change, and the most effective app functionality remains unknown. The involvement of HCPs in the use of mobile apps needs to be investigated further, needs to undergo cost-benefit analysis, and needs to be compared with the effectiveness standalone apps that do not require HCP input. Finally, a standard validated approach for medication adherence measurement is recommended for future studies to enable the comparison of findings and/or pooling of adherence data.

### Conclusions

Mobile apps appear to enhance medication adherence and improve health-related outcomes. Apps have an acceptable degree of usability; yet the app characteristics conferring usability and effectiveness are often indeterminate due to their multifactorial design. Existing evidence is currently insufficient to unreservedly recommend the use of health care apps to improve adherence to CVD medications because of the generally small sample sizes; clinical and methodological heterogeneity between studies; and disparity in app features, content, and delivery, but they may enhance medication adherence as part of a package of care.

## Appendix

Multimedia Appendix 1 Search strategy: Ovid MEDLINE (1946 to January 2020).

Multimedia Appendix 2 Medication adherence of the included randomized controlled trials.

Multimedia Appendix 3 Clinical outcomes of the included randomized controlled trials.

Multimedia Appendix 4 Meta-analysis and forest plot of the effect of app-based interventions on clinical outcomes.

## Abbreviations

BP: blood pressure
CVD: cardiovascular disease
DBP: diastolic blood pressure
HCP: health care professional
LDL-C: low-density lipoprotein cholesterol
mHealth: mobile health
PAM: patient activation measure
PRISMA: Preferred Reporting Items for Systematic Reviews and Meta-Analyses
QoL: quality of life
RCT: randomized controlled trial
SBP: systolic blood pressure
TC: total cholesterol
