# Supplementary material for: Mobile Apps to Improve Medication Adherence in Cardiovascular Disease: Systematic Review and Meta-analysis
Source: J Med Internet Res. 2021 May 25;23(5):e24190. doi: 10.2196/24190 (PMC8188316; doi:10.2196/24190)
Supplement: Multimedia Appendix 1 [file jmir_v23i5e24190_app1.pdf]

Multimedia Appendix 1. Search strategy: Ovid MEDLINE (1946 to January 2020).

1. exp Cardiovascular diseases/
2. Cardiovascular disease*.mp.
3. Cardiovascular disease$.tw.
4. 1 or 2 or 3
5. ((cell$ or mobile$) adj3 phone$).tw.
6. (handheld$ or hand-held$).tw.
7. PDA.tw.
8. (personal$ adj3 digital$).tw.
9. ("Palm OS" or "Palm Pre classic").tw.
10. (smartphone$ or smart-phone$).tw.
11. Blackberry.tw.
12. Nokia.tw.
13. (windows adj3 (mobile$ or phone$)).tw.
14. INQ.tw.
15. HTC.tw.
16. Sidekick.tw.
17. Android.tw.
18. iPhone$.tw.
19. iPad.tw.
20. iPod.tw.
21. (tablet adj3 (device$ or comput$)).tw.
22. (mhealth or m-health or "m health").tw.
23. "mobile health".tw.
24. exp Telemedicine/
25. (telehealth$ or tele-health$).tw.
26. telecare$ or tele-care$).tw.
27. (e-health or ehealth or "e health").tw.
28. (app$ adj3 (smartphone$ or smart-phone or mobile$ or phone$)).tw.
29. (palm$ adj3 computer$).tw.
30. 5 or 6 or 7 or 8 or 9 or 10 or 11 or 12 or 13 or 14 or 15 or 16 or 17 or 18 or 19 or 20 or 21 or 22 or 23 or 24 or 25 or 26 or 27 or 28 or 29
31. exp Medication Adherence/
32. exp Patient Compliance/
33. "Treatment Adherence and Compliance".tw.
34. (Treatment Adherence and Compliance).tw.
35. 31 or 32 or 33 or 34
36. 4 and 30 and 35
