# Supplementary material for: Mobile Apps to Improve Medication Adherence in Cardiovascular Disease: Systematic Review and Meta-analysis
Source: J Med Internet Res. 2021 May 25;23(5):e24190. doi: 10.2196/24190 (PMC8188316; doi:10.2196/24190)
Supplement: Multimedia Appendix 2 [file jmir_v23i5e24190_app2.pdf]

Multimedia Appendix 2. Medication adherence of the included randomized controlled trials.

Source	Total length of intervention	Medications monitoring	Medication adherence measurement	Medication adherence results
Brath et al [19]	20 weeks<br>(5 months)	Antihypertensive medication (ramipril)<br>Antihyperglycemic medication (metformin)<br>Lipid-lowering medication (simvastatin, rosuvastatin)	Intervention: remote mAMS[a]<br>Control: pill count of standard medication blisters	Intervention: median (IQR)[b], min, max:<br>Metformin 1(1–1),0.93,1<br>Simvastatin 1(1–1),0.93,1<br>Rosuvastatin 1(1–1), 0.97,1<br>Ramipril 1(1–1),0.96,1<br>Control:<br>Metformin 1(1–1),0.89,1<br>Simvastatin 1(1–1),1,1<br>Rosuvastatin 1(1–1),0.91,1<br>Ramipril 1(1–1),0.93,1
Chandler et al [20]	9 months	Antihypertensive medications	Intervention and control: 8-MMAS[c]	Intervention: mean (SD)[d]<br>Baseline: 6.83 (1.99)<br>1 month: 9.42 (0.95)<br>3 months: 9.13 (1.50)<br>6 months: 10.22 (0.43)<br>9 months: 9.81 (1.31)<br>Control:<br>Baseline: 6.99 (2.39)<br>1 month: 7.85 (1.66)<br>3 months: 7.98 (1.95)<br>6 months: 7.48 (1.7)<br>9 months: 6.84 (1.52)
Fang and Li [21]	6 months	Lipid-lowering medications (statin)	Intervention and control: 4-MMAS[e]	Intervention: mean (SD)<br>Baseline:<br>2.88 (0.71)<br>2.86 (0.71)<br>Control:<br>Baseline: 2.86 (0.87)<br>6 months: logistic regression analysis results

Source	Total length of intervention	Medications monitoring	Medication adherence measurement	Medication adherence results
Frias et al [22]	12 weeks<br>(3 months)	Antihypertensive medications (lisinopril, losartan, amlodipine)<br>Antihyperglycemic medications (metformin, glipizide)<br>Lipid-lowering medications (atorvastatin)	Intervention: DMO[f]; mean ingestion adherence	Intervention: % of the ingestion adherent<br>Baseline: 86%<br>3 months: 84%<br>Control: not assessed
Goldstein et al [23]	28 days<br>(1 month)	Not specified	Intervention: electronic self-report<br>Control: pillbox bin openings	Intervention: % (SD) adherent patients<br>1 month:<br>Overall: 76% (36)<br>Active arm: 73% (39)<br>Passive arm: 79% (33)<br>Control:<br>1 month:<br>Overall: 80% (33)<br>Active arm: 84% (32)<br>Passive arm: 76% (33)
Guo et al [24]	3 months	Oral anticoagulants	Intervention and control: 3-item Adherence Estimator: Low risk= score of 0, moderate risk= score of 2-7, and high risk=score of 8-36	Intervention:<br>Baseline: 4 (4-11)<br>1 month: 0 (0-4)<br>3 months: 2 (0-4)<br>Control:<br>Baseline: 4 (4-11)<br>1 month: 4 (0-11)<br>3 months: 4 (0-11)
Johnston et al [25]	6 months	Oral anticoagulants (ticagrelor)	Intervention and control: patient self-reported questionnaire (for non-adherence measure)	Intervention: mean (SD)<br>6 months: 16.6 (42.9)<br>Control:<br>6 months: 22.8 (41.3)

Source	Total length of intervention	Medications monitoring	Medication adherence measurement	Medication adherence results
Kim et al [26]	6 months	Antihypertensive medications	Intervention and control: 4-MMAS	Intervention: mean (SD)<br>Baseline: 6.6 (1.4)<br>6 months: 6.7 (1.4)<br>Control:<br>Baseline: 6.3 (1.4)<br>6 months: 6.5 (1.5)
Labovitz et al [27]	12 weeks<br>(3 months)	Oral anticoagulants (warfarin, dabigatran, rivaroxaban, or apixaban)	Intervention and control: pill counts and plasma samples (plasma samples were marked as adherent if drug concentration levels were above the minimum required therapeutic range (Cmin)	For all OACs[g], 3 months: based on pill count, mean% (SD)<br>Intervention: 97.2% (4.4)<br>Control: 90.6% (5.8)<br>Based on plasma samples:<br>Intervention:100%<br>Control: 50%<br>For DOACs[h],<br>3 months: based on pill count, mean (SD)<br>Intervention: 96.4 (5.1)<br>Control: 90.9 (6.0)<br>Based on plasma samples:<br>Intervention:100%<br>Control: 33%
Liu et al [28]	12 weeks<br>(3 months)	Lipid-lowering medications	Intervention and control: 4-MMAS	Intervention: % adherent patients<br>Baseline: 43.33%<br>3 months: 82.14%<br>Control:<br>Baseline: 30.00%<br>3 months: 37.93%

Source	Total length of intervention	Medications monitoring	Medication adherence measurement	Medication adherence results
Márquez Cantreras et al [29]	12 months	Antihypertensive medications	Intervention and control: MEMSs[i]	Intervention: % global adherence (95% CI) [j]<br>6 months: 93.15% (87.36 to 98.94)<br>12 months: 91.78% (85.48 to 98.08)<br>Control:<br>6 months: 74.66% (64.7 to 84.6)<br>12 months: 62.66% (51.26 to 73.14)
Mertens et al [30]	28 days<br>(1 month)	Not specified	Intervention and control: A14-scale questionnaire: 5-point Likert scale, ranging from "never" (0) to "very often" (4), based on the A14 scale, values <50 as nonadherent, and values 50-56 as adherent	Intervention: mean (SD)<br>Baseline: 50.02 (3.44)<br>1 month: 53.96 (2.01)<br>Control:<br>Baseline: 50.02 (3.44)<br>1 month: 52.60 (2.49)
Morawski et al [31]	12 weeks<br>(3 months)	Antihypertensive medications (thiazide, calcium channel blocker, b-blocker, angiotensin converting enzyme inhibitor, or angiotensin receptor blocker)	Intervention and control: 8-MMAS	Intervention: mean (SD)<br>Baseline: 6.0 (1.8)<br>3 months: 6.3 (1.6)<br>Control:<br>Baseline: 5.7 (1.8)<br>3 months: 5.7 (1.8)

Source	Total length of intervention	Medications monitoring	Medication adherence measurement	Medication adherence results
Ni et al [32]	30 days (1 month)	Cardiovascular medications	Intervention and control: 3-question Voils Medication Non-adherence Extent Scale	Intervention: mean (SD)<br>Baseline: 6.28 (2.14)<br>1 month: −1.35 (2.18)<br>Control:<br>Baseline: 6.28 (1.99)<br>1 month: −0.69 (1.58)
Santo et al [33]	3 months	Cardiovascular medications	Intervention and control: 8-MMAS	Intervention: mean (SD)<br>Baseline: 6.8 (1.40)<br>3 months: 7.11 (1.05)<br>Control:<br>Baseline: 7.0 (1.29)<br>3 months: 6.63 (1.04)
Sarfo et al [34]	3 months	Antihypertensive medications	Intervention and control: mean MPR[k] and 8-MMAS	Intervention: mean (SD)<br>Baseline: 10.3 (3.1)<br>9 months: 13.0 (1.5)<br>MPR:<br>3 months: 0.88 (0.40)<br>9 months: 0.95 (0.16)<br>Control:<br>Baseline: 11.5 (2.8)<br>9 months: 13.0 (1.7)<br>MPR:<br>3 months: 0.64 (0.45)<br>9 months: 0.98 (0.24)

[a]mAMS: medication adherence measurement system.

[b]IQR: interquartile range.

[c]8-MMAS: 8-item Morisky Medication Adherence Scale.

[d]SD: standard deviation.

[e]4-MMAS: 4-item Morisky Medication Adherence Scale.

[f]DMO: digital medicine offering system.

[g]OACs: oral anticoagulants.

[h]DOACs: direct oral anticoagulants.

[i]MEMSs: Medication Event Monitoring Systems.

[j]95% CI: 95% confidence interval.

[k]MPR: medication possession ratio.
