# Supplementary material for: Mobile Apps to Improve Medication Adherence in Cardiovascular Disease: Systematic Review and Meta-analysis
Source: J Med Internet Res. 2021 May 25;23(5):e24190. doi: 10.2196/24190 (PMC8188316; doi:10.2196/24190)
Supplement: Multimedia Appendix 3 [file jmir_v23i5e24190_app3.pdf]

Multimedia Appendix 3. Clinical outcomes of the included randomized controlled trials.

Source	Total length of intervention	Blood pressure	Cholesterol level	Blood glucose level
Brath et al [19]	20 weeks<br>(5 months)	Intervention and control (crossover design): mean<br>Baseline:<br>SBP[a] 133<br>DBP[b] 75<br>5 months:<br>SBP 128<br>DBP 70	Intervention and control (crossover design): median (lower-upper quartile)<br>Baseline:<br>TC[c] 166 (147-183) mg/dL<br>LDL-C[d] 87 (68-102) mg/dL<br>HDL-C[e] mean (SD)[f]<br>48 (12) mg/dL<br>5 months:<br>TC 155 (141-167) mg/dL<br>LDL-C 80 (67-92) mg/dL<br>HDL-C 47 (13) mg/dL	Intervention and control (crossover design):<br>HbA_{1c}[g], %: mean (SD)<br>Baseline:<br>7.2 (0.8)<br>5 months:<br>7.1 (0.9)
Chandler et al [20]	9 months	Intervention: mean<br>Baseline:<br>SBP 150.7, DBP 86.8<br>1 month:<br>SBP 125.3, DBP 82.1<br>3 months:<br>SBP 120.4, DBP 71.3<br>6 months:<br>SBP 121.2, DBP 74.7<br>9 months:<br>SBP 121.8, DBP 74.2<br>Control:<br>Baseline:<br>SBP 152.3, DBP 84.6<br>1 months:<br>SBP 140.6, DBP 78.3<br>3 months:<br>SBP 137.5, DBP 75.4<br>6 months:<br>SBP 138.9, DBP 77.5<br>9 months:<br>SBP 145.7, DBP 79.4	Not assessed	Not assessed

Source	Total length of intervention	Blood pressure	Cholesterol level	Blood glucose level
Frias et al[h] [22]	12 weeks<br>(3 months)	Intervention: mean (SD)<br>Mean (SE)[i]<br>Baseline:<br>SBP 149.3 (SE 1.5)<br>=149.3 (SD 13.42)<br>DBP 86.2 (SE 3.2)<br>=86.2 (SD 28.62)<br>3 months: mean change (SD)<br>SBP −20.9 (SE 3.4)<br>=−20.9 (SD 30.41)<br>DBP −8.6 (SE 2.2)<br>=−8.6 (SD 19.68)<br>Control:<br>Baseline:<br>SBP 155.4 (SE 3.0)<br>=155.4 (SD 16.16)<br>DBP 83.9 (SE 2.9)<br>=83.9 (SD 15.62)<br>3 months: mean change (SD)<br>SBP −15.2 (SE 2.0)<br>=−15.2 (SD 10.77)<br>DBP −5.8 (SE 2.2)<br>=−5.8 (SD 11.85)	Intervention: mean (SD)<br>Baseline:<br>TC 177.4 (SE 9.5) mg/dL<br>=4.59 (SD 1.57) mmol/L<br>LDL-C 103.9 (10.1 SE) mg/dL<br>=2.69 (SD 1.67) mmol/L<br>3 months:<br>TC −29.5 (SE 7.4) mg/dL<br>=−0.76 (SD 1.23) mmol/L<br>LDL-C −21.3 (SE 10.0) mg/dL<br>=−0.55 (SD 1.66) mmol/L<br>Control:<br>Baseline:<br>TC 174.4 (SE 13.1) mg/dL<br>=4.51 (SD 1.62) mmol/L<br>LDL-C 99.3 (SE 6.7) mg/dL<br>=2.57 (SD 0.83) mmol/L<br>TC −21.9 (SE 10.2) mg/dL<br>=−0.57 (SD 1.27) mmol/L<br>LDL-C −9.5 (SE 5.6) mg/dL<br>=−0.25 (SD 0.69) mmol/L	Intervention:<br>HbA_{1c}, %: mean (SD)<br>Baseline:<br>8.66 (SE 0.18)<br>=8.66 (SD 1.61)<br>3 months:<br>−0.19 (SE 0.14)<br>=−0.19 (SD 1.25)<br>Control:<br>Baseline:<br>8.28 (SE 0.38)<br>=8.28 (SD 2.05)<br>3 months:<br>0.26 (SE 0.35)<br>=0.26 (SD 1.88)

Source	Total length of intervention	Blood pressure	Cholesterol level	Blood glucose level
Johnston et al [25]	6 months	Intervention: mean (SD)<br>Baseline:<br>SBP 131.1 (14.6)<br>DBP 78.8 (11.0)<br>3 months: mean change (SD)<br>SBP −3.1 (18.7)<br>DBP −1.2 (13.1)<br>6 months:<br>SBP −0.6 (16.3)<br>DBP 1.9 (13.0)<br>Control:<br>Baseline<br>SBP 125.2 (17.9)<br>DBP 75.5 (11.0)<br>3 months:<br>SBP 3.0 (16.2)<br>DBP 2.0 (10.5)<br>6 months:<br>SBP −1.1 (19.6)<br>DBP 1.5 (11.8)	Intervention: mean (SD)<br>Baseline:<br>LDL-C 3.9 (1.2) mmol/L<br>3 months:<br>LDL-C −1.9 (1.1) mmol/L<br>6 months:<br>LDL-C −1.8 (1.0) mmol/L<br>Control:<br>Baseline:<br>LDL-C 3.3 (0.9) mmol/L<br>3 months:<br>LDL-C −1.4 (1.1) mmol/L<br>6 months:<br>LDL-C −1.0 (1.5) mmol/L	Not assessed
Kim et al [26]	6 months	Intervention: mean (SD)<br>Baseline:<br>SBP 136.1 (15.2)<br>DBP 86.3 (12.8)<br>6 months:<br>SBP 133.4 (12.9)<br>DBP 82.8 (11.2)<br>Control:<br>Baseline:<br>SBP 145.9 (19.5)<br>DBP 93.1 (14.1)<br>6 months:<br>SBP 140.2 (18.4)<br>DBP 85.3 (12.1)	Not assessed	Not assessed

Source	Total length of intervention	Blood pressure	Cholesterol level	Blood glucose level
Liu et al [28]	12 weeks<br>(3 months)	Not assessed	Intervention: mean (SD)<br>Baseline:<br>TC 3.91 (0.80) mmol/L<br>TG[j] 1.63 (0.78) mmol/L<br>LDL-C 2.29 (0.69) mmol/L<br>3 months:<br>TC 3.61 (0.82) mmol/L<br>TG 1.36 (0.59) mmol/L<br>LDL-C 2.18 (0.73) mmol/L<br>Control:<br>Baseline:<br>TC 4.44 (1.13) mmol/L<br>TG 1.74 (0.84) mmol/L<br>LDL-C 2.68 (1.16) mmol/L<br>3 months:<br>TC 4.39 (0.65) mmol/L<br>TG 2.11 (1.05) mmol/L<br>LDL-C 2.56 (0.74) mmol/L	Not assessed
Márquez Cantreras et al [29]	12 months	Intervention: mean (SD)<br>Baseline:<br>SBP 134.7 (14)<br>DBP 81.64 (8)<br>6 months:<br>SBP 130.6 (12)<br>DBP 78.76 (8)<br>12 months:<br>SBP 132.2 (12)<br>DBP 78.5 (7)<br>Control:<br>Baseline:<br>SBP 134.47 (8)<br>DBP 81.9 (6.8)<br>6 months:<br>SBP 135.2 (8)<br>DBP 82.2 (8)<br>12 months:<br>SBP 134.4 (11)<br>DBP 81.4 (9)	Not assessed	Not assessed

Source	Total length of intervention	Blood pressure	Cholesterol level	Blood glucose level
Morawski et al [31]	12 weeks<br>(3 months)	Intervention: mean (SD)<br>Baseline:<br>SBP 151.4 (9.0)<br>3 months:<br>SBP 140.8 (15.7)<br>Control:<br>Baseline:<br>SBP 151.3 (9.4)<br>3 months:<br>SBP 141.2 (17.3)<br>DBP not assessed	Not assessed	Not assessed
Ni et al [32]	30 days<br>(1 month)	Intervention: mean (SD)<br>Baseline:<br>SBP 125.7 (11.80)<br>DBP 74.24 (10.07)<br>1 month: mean change (SD)<br>SBP 0.93 (10.40)<br>DBP 0.81 (10.38)<br>Control:<br>Baseline:<br>SBP 123.60 (14.27)<br>DBP 73.78 (8.45)<br>1 month: mean change (SD)<br>SBP −3.76 (25.72)<br>DBP 8.71 (21.43)	Not assessed	Not assessed

Source	Total length of intervention	Blood pressure	Cholesterol level	Blood glucose level
Santo et al [33]	3 months	Intervention: mean (SD)<br>Baseline:<br>SBP 125.9 (17.32)<br>DBP 80.3 (10.51)<br>3 months:<br>SBP 125.0 (17.05)<br>DBP 80.2 (8.76)<br>Control:<br>Baseline:<br>SBP 124.4 (20.17)<br>DBP 78.9 (11.42)<br>3 months:<br>SBP 126.0 (19.12)<br>DBP 81.1 (10.47)	Intervention: mean (SD)<br>Baseline:<br>TC 4.1 (1.12) mmol/L<br>LDL-C 2.2 (0.99) mmol/L<br>3 months:<br>TC 3.60 (0.90) mmol/L<br>LDL-C 1.62 (0.75) mmol/L<br>Control:<br>Baseline:<br>TC 4.1 (1.17) mmol/L<br>LDL-C 2.2 (0.94) mmol/L<br>3 months:<br>TC 3.60 (0.84) mmol/L<br>LDL-C 1.68 (0.70) mmol/L	Not assessed
Sarfo et al [34]	3 months	Intervention: mean (SD)<br>Baseline:<br>SBP 141.3 (30.3)<br>DBP 91.4 (18.0)<br>3 months:<br>SBP 137.3 (21.4)<br>DBP 89.1 (15.0)<br>Control:<br>Baseline:<br>SBP 146.3 (22.5)<br>DBP 89.6 (12.9)<br>3 months:<br>SBP 142.0 (26.5)<br>DBP 85.0 (14.0)	Not assessed	Not assessed

[a]SBP: systolic blood pressure.

[b]DBP: diastolic blood pressure.

[c]TC: total cholesterol.

[d]LDL-C: low-density lipoprotein cholesterol.

[e]HDL-C: high-density Lipoprotein cholesterol.

[f]SD: standard deviation.

[g] HbA_{1c}: glycated haemoglobin.

[h]The conversion factor of LDL-C to mmol/L, multiply values by 0.02586.

[i]SE: standard error.

[j]TG: triglyceride.
