# Supplementary material for: Mobile Apps to Improve Medication Adherence in Cardiovascular Disease: Systematic Review and Meta-analysis
Source: J Med Internet Res. 2021 May 25;23(5):e24190. doi: 10.2196/24190 (PMC8188316; doi:10.2196/24190)

Multimedia Appendix 4. Meta-analysis and forest plot of the effect of app-based interventions on clinical outcomes.

Figure 1. Meta-analysis and forest plot of the effect of app-based interventions on systolic blood pressure (SBP), using mean values (A), using mean change values (B). REML: restricted maximum likelihood.

(A)

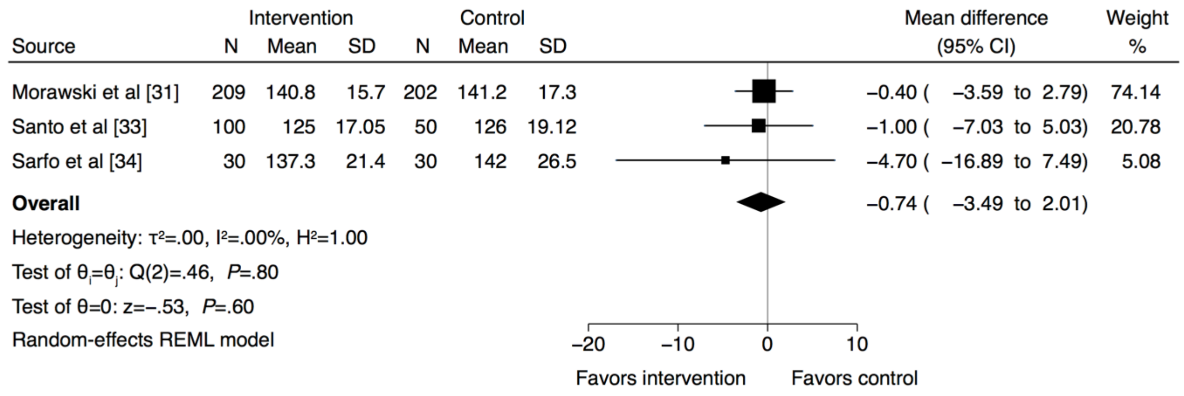

(B)

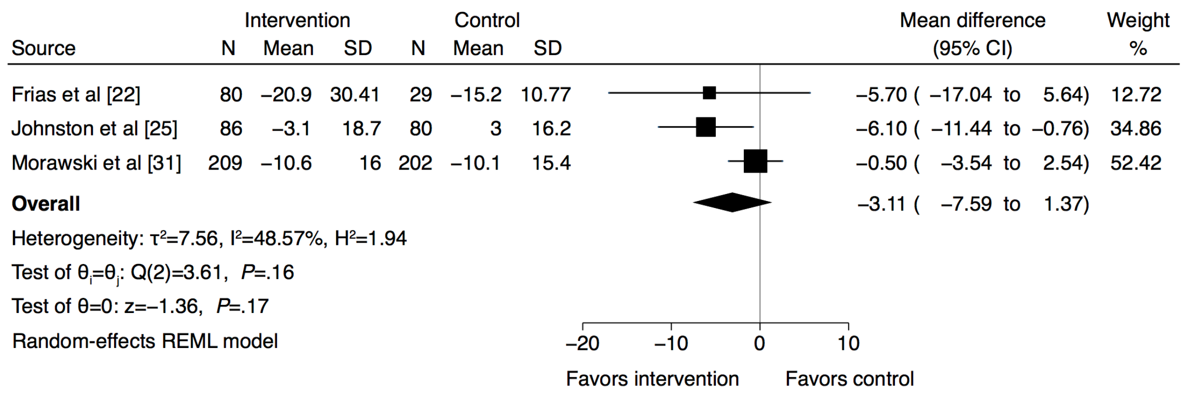

Figure 2. Meta-analysis and forest plot of the effect of app-based interventions on diastolic blood pressure (DBP), using mean values (A), using mean change values (B). REML: restricted maximum likelihood.

(A)

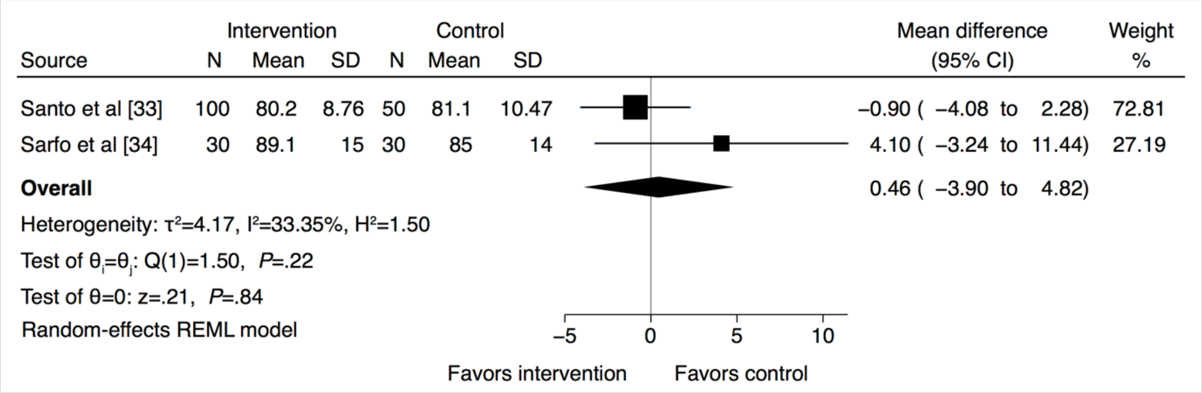

(B)

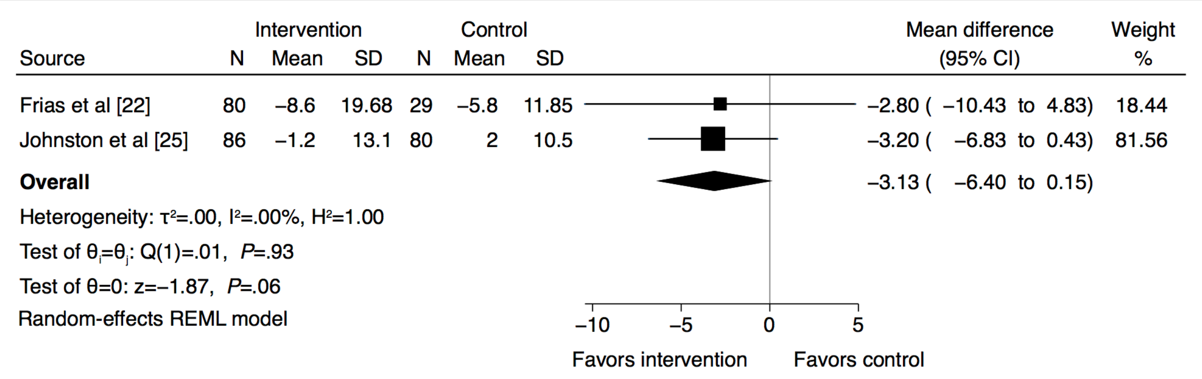

Figure 3. Meta-analysis and forest plot of the effect of app-based interventions on low-density lipoprotein cholesterol (LDL-C), using mean values (A), using mean change values (B). REML: restricted maximum likelihood.

(A)

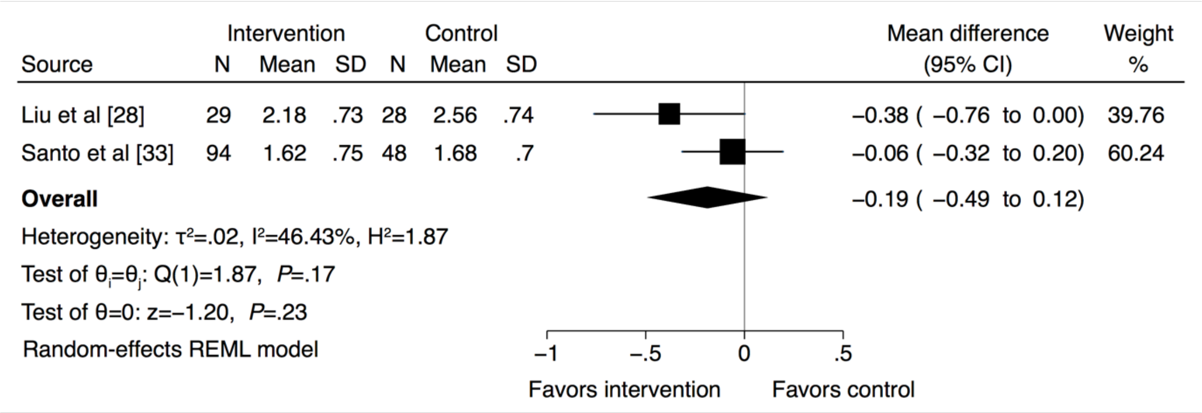

(B)

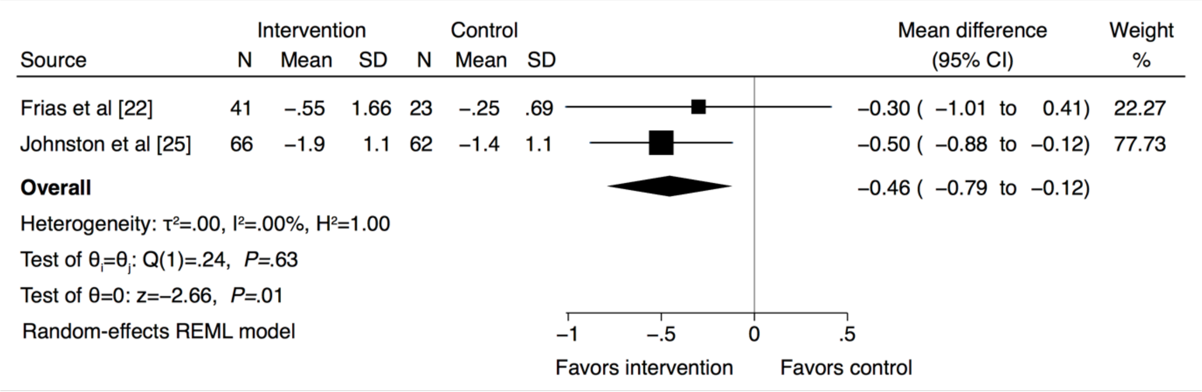

Figure 4. Meta-analysis and forest plot of the effect of app-based interventions on total cholesterol (TC). REML: restricted maximum likelihood.

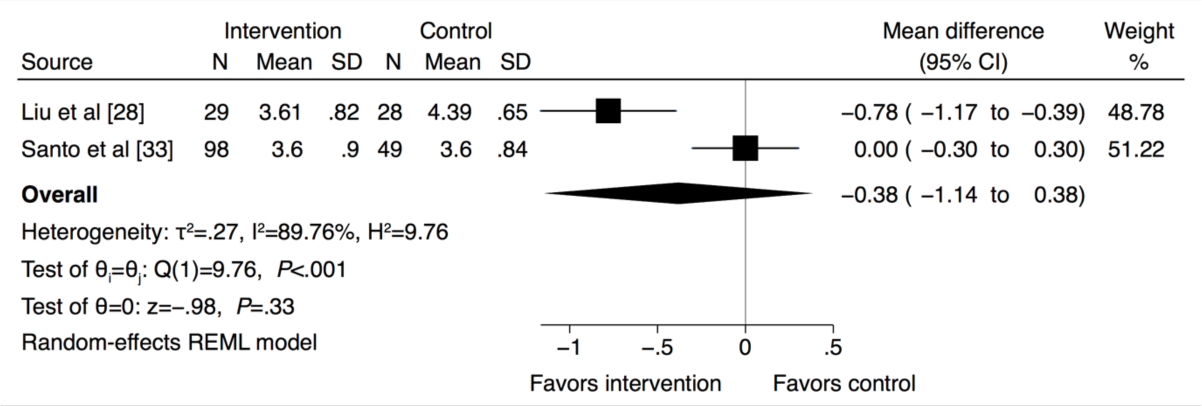

Supplement: Multimedia Appendix 4 [file jmir_v23i5e24190_fig.pdf]
